# Assessing the Effects of Tourist Provisioning on the Health of Wild Barbary Macaques in Morocco

**DOI:** 10.1371/journal.pone.0155920

**Published:** 2016-05-20

**Authors:** Laëtitia Maréchal, Stuart Semple, Bonaventura Majolo, Ann MacLarnon

**Affiliations:** 1 Department of Life Sciences, University of Roehampton, London, United Kingdom; 2 School of Psychology, University of Lincoln, Lincoln, United Kingdom; Sichuan University, CHINA

## Abstract

Feeding wildlife is a very popular tourist activity, largely because it facilitates the close observation of animals in their natural habitat. Such provisioning may benefit animals by improving their survival and reproductive success, especially during periods of natural food shortage. However, provisioning by tourists may also have negative impacts on the health of the animals involved; to date such impacts are poorly understood. Here, we investigated the effects of tourist provisioning on the health of wild adult Barbary macaques, *Macaca sylvanus*, in Morocco. We compared health measures between a heavily provisioned group and a group that received negligible food from tourists and, in the former group, we also assessed health measures in relation to the intensity of provisioning. We used a broad range of non-invasive health measures relating to birth rate and survival, disease and injury risk, body size and condition, and physiological stress. Our findings indicate that feeding by tourists may overall have negative impacts on the health of Barbary macaques, being linked in particular to larger body size, elevated stress levels and more alopecia. Finally, we propose a framework to help consider the potential costs and benefits of provisioning, which may facilitate future research and management decisions on whether—and how much—provisioning is acceptable.

## Introduction

Feeding wildlife is popular with tourists, particularly as it attracts animals for close viewing [1]. While tourist provisioning may increase survival and rate of reproduction (e.g. Japanese macaques, *Macaca fuscata* [2]), in recent years there has been growing concern about the potential negative effects of provisioning on the health of wild animals [1, 3, 4]. Tourism is increasingly viewed as a potentially powerful tool for conservation, because it can generate funds and raise awareness of conservation issues [5, 6]. However, this activity is often associated with wildlife tourists feeding animals (e.g. [1]; Caribbean reef sharks, *Carcharhinus perezi* [7]; Northern Bahamian Rock Iguanas, *Cyclura cychlura* [8]), even when such feeding is discouraged or expressly forbidden (e.g. Barbary macaques in Gibraltar: [9]; southern stingrays, *Dasyatis Americana* [10]; Bottlenose dolphin, *Tursiops sp*. [11]). Therefore, assessing whether and how tourist provisioning affects animal health is important if wildlife tourism is to be used as a tool for conservation.

Health is linked directly to fitness and quantifying the health impacts of tourist provisioning for the animals involved is therefore important for informing conservation management and for the development and implementation of associated regulations. Consequently, health assessment can be a useful tool for conservation management, without the requirement for the longer term study typically needed, at least in larger mammals, to acquire indices of fitness such as birth rates, survival or reproductive success. For health assessment to be effective in this way, readily applied measures are needed [12, 13]. The development of a range of non-invasive techniques has recently facilitated researchers’ attempts to investigate the impacts of human disturbance on the health of wildlife.

Studies have provided evidence that provisioning is linked to an increase in risk of injury, such that quantification of visible injuries can provide a useful measure of health impact (Tibetan macaques, *Macaca thibetana* [14]; southern stingrays [15]). Photogrammetry has been used to investigate the impacts of provisioning on body size in Barbary macaques, with tourist fed animals found to have a larger body size than wild feeding individuals [16]; if such animals are overweight or even obese—as has also been suggested in the case of tourist provisioned Japanese macaques [17] and Barbary macaques in Gibraltar [18]—this may lead to diet-related disease and disorders, such as diabetes, cardiovascular diseases, and reduced fertility [19–21].

Visual assessment of coat condition has provided evidence that exposure to tourism is associated with poorer body condition in ring tailed lemurs, *Lemur catta* [22] and Barbary macaques [16]. Evidence has also been found that the presence of tourists increases parasite burden in a range of species including stringrays [10], common wall lizards, *Podarcis muralis* [23], chimpanzees, *Pan troglodytes* [24] and mountain gorillas, *Gorilla gorilla beringei* [25]. Finally, tourism has been associated with increased stress hormone levels in Magellanic penguins, *Spheniscus magellanicus* [26], Barbary macaques [27] and western lowland gorillas, *Gorilla gorilla gorilla* [28].

Studies exploring the effects of tourism on the health of animals have tended to focus on one or a few health measures, limiting our understanding of the full spectrum of such effects. Tourist provisioning in particular might affect multiple dimensions of animal health. Investigating a broad, multifaceted range of measures will improve our understanding of the full impacts of provisioning on the health of the animals involved. In this study, we aimed to investigate the impacts of tourist provisioning on the health of adult Barbary macaques in Ifrane National Park, Middle Atlas Mountains, Morocco, using a broad battery of measures: birth and mortality rates were used to explore short-term population level effects; number of scars and injuries, lameness and symptoms of illness were used to assess physical health; body size, coat quality and levels of alopecia were used to assess general body condition; and faecal glucocorticoid levels were used to evaluate physiological stress levels. We used two complementary methods of analysis: first we compared the different health measures between two groups of Barbary macaques, one experiencing daily provisioning by tourists, and another one relying on natural food resources; second, we explored the relationships between the different health measures and the intensity of provisioning in the provisioned group.

Barbary macaques are found in mountainous regions of Morocco and Algeria [29, 30], and recently fewer than 10,000 individuals were estimated to remain in these areas [31]. The species was therefore classified in 2008 as Endangered by IUCN [32]. In 2012, the *Conservation Action Plan for the Barbary Macaque in Morocco* was initiated [33], and as part of the different actions proposed, the development of primate tourism was encouraged. However, such tourism in Morocco is currently unregulated, and feeding monkeys is a common practice. If primate tourism is to work as a tool for conservation of Barbary macaques, investigation of the impacts of provisioning on their health is urgently needed. Such studies can be used to inform management decisions and guide the regulation of tourist provisioning.

## Materials and Methods

### Study sites and animals

**Fig 1:**
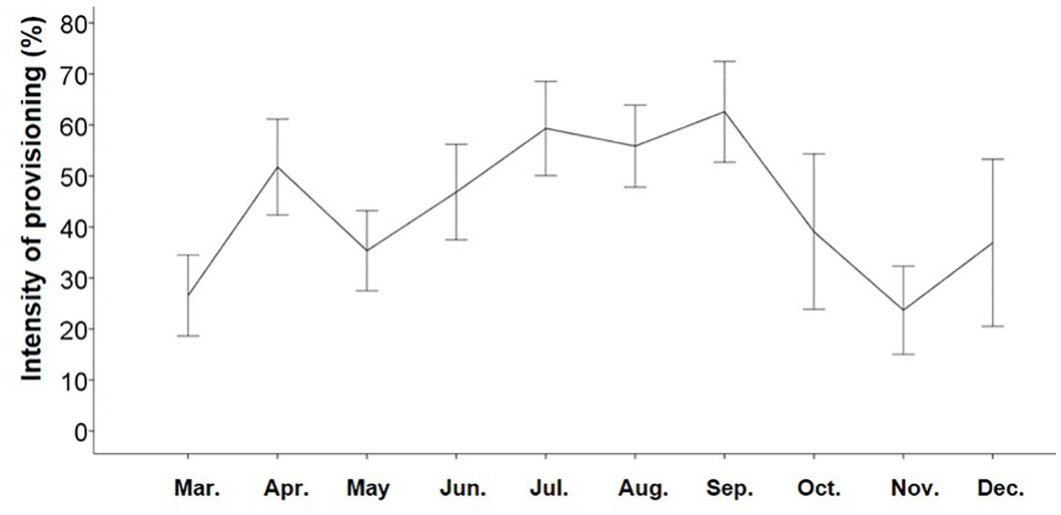
Graph of the intensity of provisioning (%) from March to December 2012. Intensity of provisioning represents the percentage of their feeding activity during which macaques were observed eating food provided by humans.

This study was conducted at two sites approximately 2km apart in Ifrane National Park, in the Middle Atlas Mountains of Morocco. At the first study site (33°25.0N; 005°10.0W), a group of Barbary macaques (the Tourist Group: TG) experiences high daily tourist numbers and a large amount of provisioning. Provisioning intensity in TG varies across the year (Fig 1), with macaques spending on average 44.6% of their feeding activity eating food provided by humans. Tourists have been visiting monkeys at this site for over ten years. At the second study site (33°23.695N; 005°11.948W), another group of Barbary macaques (the Green Group: GG) very rarely encounter tourists, and they rely on natural food resources. The two study sites are very close to each other and share the same habitat type; natural food availability varies across the year, being low in summer and winter, and more abundant in spring and autumn [34].

At the start of data collection, the Tourist Group was composed of 40 individuals: 12 adult males, 12 adult females, 2 sub-adult males, 1 sub-adult female, 6 juveniles, and 7 one-year-old infants; in addition, 5 infants were born during the study period. The Green Group was composed of 24 individuals at the start of data collection: 6 adult males, 6 adult females, 1 sub-adult male, 6 juveniles, and 5 one-year-old infants; 6 infants were born during the study period. Both study groups fall within the normal group size range (13–88 individuals) reported in this species [35]. Data were collected on 17 adult individuals from the Tourist Group (8 males and 9 females) and 11 adults from the Green Group (5 males and 6 females); sub-adult and young adults (3 males and 2 females in TG, and 1 male in GG) were excluded from data collection because they had not reached their full body size. Two adults from the Tourist Group were also excluded from main data collection: one female that disappeared at the start of the study period, and one male that died in April 2012.

### Data collection

Research permission was provided by the Haut-Commissariat aux Eaux et Forêts et à la Lutte Contre la Désertification of Morocco (Number 235). Data were collected by LM and a team of field assistants, from March 2012 to December 2012, with a total of 207 days for Tourist Group (5 days/week), and 79 days for Green Group (2 days/week), during which each group was monitored for approximately 9 h per day from 8 am to 5 pm. Hourly behavioural scan sampling [36] was used to record the type of food being eaten (human vs. natural food, or none) by each visible macaque. We used ten distinct non-invasive measures to assess the health of the study animals, detailed below.

#### Births and deaths

Births were recorded when a female was first observed taking care of a new-born infant. The number of births was recorded for both groups in the study period and combined with additional data collected on a similar basis for 2011 and 2013. Deaths were recorded in the study period, either when a body was found or when an animal had disappeared for more than two months and had not been seen in neighbouring groups.

#### Scars, injuries, lameness and disease symptoms

The presence of new scars and the occurrence of any lameness were recorded once a month for each animal. Data on injuries and disease symptoms, such as coughing, sneezing and diarrhoea, were recorded *ad libitum* on each observation day.

#### Body size

**Fig 2:**
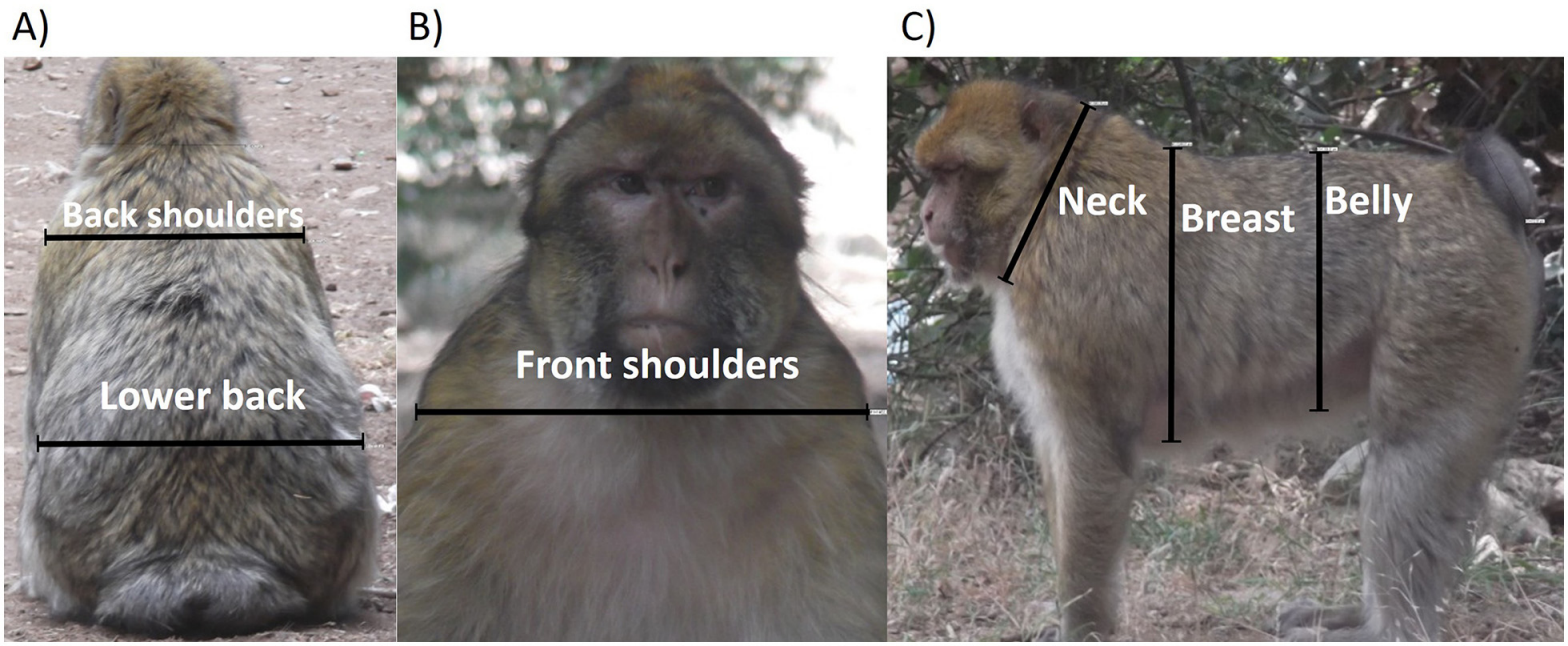
Examples of photogrammetry measurements for each body dimension measured. a) back sitting posture, b) front sitting posture, c) side standing posture.

The term body size in this study is used to denote a measure of the overall size of the study animals, and does not refer specifically to body fatness or bone length *per se*. Photogrammetry was used to determine the body size of each individual, following procedures used by [16, 37, 38]. We aimed to take one set of pictures per month per individual in three different postures (back sitting, front sitting and side standing) to measure six body dimensions: back shoulders and lower back (from back sitting posture); front shoulders (from back sitting posture); neck, belly and breast (from side standing posture) (see Fig 2). All measurements were made as straight lines. To ensure that each measurement was taken from the same end points, we compared several pictures of the same individual.

Digital photos were taken using a digital Fujifilm camera (Finepix T200); Fujinon lens f = 5–50 mm 1: 3.4–5.6. For the camera calibration, the resolution of the image size was Large 1,056 frames, 14 megapixels (4288x3216), picture-taking mode was ‘natural light’ and optical zoom was full (x 10) meaning the focal lens length was constant (50mm). Distances between camera and the subject were measured with a Bosch DLE 40 Laser Measurer, class 2. Different measures of the size of the monkey (Fig 2), in pixels, were extracted using tpsDIG2 software [39]. The criteria for picture inclusion were: total visibility of the primate body, a sharp image taken at 5–15 m from the subject at the monkey’s height, subject perpendicular to the camera frame (following [16]).

The conversion factor for each image was determined by measuring an object of known size at different distances, using the methods and camera settings as described above. The conversion factor (c) was determined as: c = object size (cm) / [Distance from camera (m) x Size of object in image (pixels)]. An average conversion factor of 0.00297 was obtained, and used to convert the sizes of different body dimensions: body dimension (cm) = distance from camera (m) x size of dimension in image (pixels) x 0.00297. The average measurement error (+/-0.33cm) was determined by comparing the actual size of the object and the size of the object obtained using the photogrammetric method. The reliability of the photogrammetric technique was determined by calculating the coefficient of variation (CV) of repeated estimations of an animal‘s body dimensions from the same photograph, and of different pictures of the same animal taken during the same month assessed by the same observer. Coefficients of variation were 0.73% within photos, and 4.32% for the same animal within the month.

All six body dimension measurements were highly correlated, with correlation coefficients > 0.8, and therefore a principal components analysis (PCA) was run on all measurements, using one value for each individual for each month. The first principal component, PCA1, accounted for 83.2% of variance and the second PCA 2 accounted for 12.9% of variance. All variables loaded strongly and positively on PCA 1. In morphometric measurement studies, PCA 1 generally accounts for body size and PCA 2 for body shape [40]; therefore the loading on PCA 1 is referred to as body size in the following analyses.

#### Coat quality

Once a month and on the same day, two observers assessed the coat quality of each animal. A 1–4 scoring scale based on the brightness and general appearance of hair was used: (1) Very good quality fur: the quality of fur is very good on the whole body (i.e. head, back, sides, legs); bright and smooth coat with straight, thick and strong hair, (2) Good quality fur: the quality of fur is mainly very good but it is not homogenous on the whole body, (3) Poor quality fur: the quality of fur is mainly poor with some parts with good quality fur, (4) Extremely poor quality fur: dull and rough coat with hair broken, fine and weak (not seen in either group). Observations focused on the head, back, sides and legs. The fur around the stomach area was not taken into consideration, as different fur quality in that area may be unrelated to coat quality on the rest of the body [16]. Data were not collected during days when it rained, nor when the humidity was above 80%, because humidity and rainfall may affect the appearance of the fur. Coat quality was recorded when individuals had not received any grooming in the 10 min prior to the observation. An inter-observer reliability test indicated no significant difference in coat quality scores between observers (Wilcoxon matched pairs test: N = 28, z = -0.832, P = 0.405).

#### Alopecia

Alopecia, defined as aggregated hair loss revealing the skin underneath, was assessed by two observers using a visual scoring system (1–3) adapted from [41]: (1) Very good coat quality, with the whole body completely covered, (2) A few small patches of alopecia (2–5 cm^2^), (3) Large patches of alopecia (≥ 5 cm^2^), or numerous small ones totalling 25–50% of the body surface. A test of inter-observer reliability indicated no significant difference for alopecia scores (Wilcoxon matched pairs test: N = 28, z = -0.16, P = 0.414).

#### Faecal glucocorticoid metabolite (FGC) levels

We analysed 1106 faecal samples (43 samples on average per individual in the Tourist Group and 33 samples on average per individual in the Green Group). Samples were collected opportunistically; we aimed to collect one sample per individual per week for the Green Group and two samples per two weeks for the Tourist Group (one of these two samples was collected on Sunday, Monday or Tuesday to reflect physiological stress levels associated with the weekend when tourist frequency was generally higher, and one sample was collected on Wednesday, Thursday or Saturday, linked with week days when tourist frequency was generally lower).

Below we describe the data collection and enzyme-immunoassay procedure as previously explained in detail in [27]. Following [42], samples from individuals seen defecating were collected into individual tubes (Azlon 30ml HDPE; cat. BWH0030PN) which were placed in a freezer bag with ice packs in the field before transfer to a -20°C freezer at the end of each day. Frozen samples were transferred to the University of Roehampton hormone laboratory for analysis. Each sample was freeze-dried and ground to a fine powder before 0.05–0.1 mg was extracted in 3ml of 80% methanol. After vortexing for 10 min, and centrifugation for 20 min at 4500 rpm, the supernatant was separated for analysis. Extraction efficiency, determined by the recovery of tritiated oestradiol added to the samples before extraction [43], was 85.1 +/- 5.2%. We analysed faecal extracts for concentrations of cortisol metabolites using a group-specific enzyme immunoassay (EIA) for the measurement of 11ß-hydroxyetiocholanolone [44], previously validated for monitoring glucocorticoid output in Barbary macaques [45]. Assay procedures followed those described in detail by [46]. The sensitivity of the assay at 90% binding was 2.34pg/well. Intra-assay coefficients of variation, calculated from repeated measures of high and low concentration quality controls, were 5.5% (high) and 8.5% (low); inter-assay coefficients of variation were 9.5% (high) and 13% (low).

FGC concentrations were matched with the behavioural and provisioning data from two days preceding the faecal sample collection, or, where these were not available, one day preceding sample collection, taking account of an excretion lag of 24-48h [45].

### Data analysis

In the Green Group, all females gave birth; in the Tourist Group, three females gave birth and six did not. As gestation and lactation might affect the different health measures (e.g. alopecia in rhesus macaques [47]; FGC levels in yellow baboons, *Papio cynocephalus* [48], rhesus macaques [49]), where appropriate we conducted analyses considering separately females in the Tourist Group that gave birth and females in this group that did not give birth.

Due to small sample sizes, it was not possible to explore statistically if there was a difference between groups in death and birth rates, occasional disease symptoms (i.e. coughing/sneezing and diarrhoea) or lameness, so we present descriptive statistics of such events and measures for each group. An index of diarrheal symptoms was calculated for each group. This index represents the number of diarrheal events divided by the number of studied animals in the group and by the number of observation days.

Health measures were compared between groups both over the entire 10 month study period, and for each individual month of the study. Monthly comparisons were conducted because Barbary macaques live in an extreme and highly seasonal environment in which they experience marked climatic variation across the year [50], and periods of scarcity of food and water [34]. The species also shows strong breeding seasonality with associated high levels of competition and aggression in the short mating season [51]. It is possible that this seasonal variation in ecological and social stressors may differently impact the health of provisioned and non-provisioned animals, and differences between troops may therefore be missed if only measures averaged across the whole study period are taken.

Comparisons were made between provisioned females who gave birth (N = 3), those who did not give birth (N = 6), and non-provisioned females who all gave birth (N = 6); and between provisioned males (N = 8) and non-provisioned males (N = 5). For comparison between the two groups across the full study period we used either an independent sample t-test or a Mann-Whitney test. For comparisons between groups per month, we used sequential Bonferroni tests with an adjustment (k) equal to 10 to control the type I error rate [52].

We tested whether the level of provisioning of the Tourist Group animals was related to the different health measures using generalised linear mixed models (GLMM [53]) with each of the health measures as the dependent variable. As ecological and social factors may also influence the health parameters, we included monthly rainfall, birth, rank and social season as control factors (see definitions in Table 1). In summary, the level of provisioning, monthly rainfall, rank and social season were included as fixed effects, and macaque ID as a nested random effect. All models were fitted in R 2.15.0 [54] using the function lmer of the R-package lme4 [55]. The models generally have 10 or more observations per predictors, which is within the range (10–15 observations per predictor) recommended by previous research to avoid over-fitted models [56], except for some of the models for males which have on average 9.5 observations per predictor.

**Table 1:** Summary of the predictor variables and their definitions.

Predictor variables	Description
Provisioning	Percentage of total feeding scans spent feeding on human food.
Birth vs. No Birth	Females who gave birth and females who did not give birth during the study period.
Rainfall (as an indicator of food availability)	Monthly average rainfall. Rainfall is linked with natural food availability in the Barbary macaque range [35]. In the present study, monthly average body size for all individuals in the non-provisioned group was positively correlated with monthly rainfall (N = 10, r_s_ = 0.888, P = 0.001), suggesting that rainfall is an indirect measure of natural food availability.
Rank	Determined using normalised David scores, z-transformed.
Social season	Four seasons were determined: pre-birth, birth, post-birth and mating. Mating season was from the first complete copulation to last complete copulation observed. Pre-birth season was between the mating and the birth seasons. Birth season was from the first birth to the birth of the last infant. Post-birth was between birth and mating seasons.

For each model, the significance of the full final model was compared to the corresponding null model using a likelihood ratio test (R function ANOVA with argument test set to “Chisq”) [57]. The null model corresponds to the full model excluding all independent variables, which are replaced by the value 1 in the model. The significance of each individual predictor included in the model was accepted only if this likelihood ratio test was significant and assumptions were met. We used the full model to test the effects of individual predictors, and not the “best fit model”, as recommended by [58]. Using this method also enabled consistent analysis of the data, using the same model for the different dependent variables tested. In each model, collinearity between independent variables was checked in order to avoid including within the same model two independent variables that were highly correlated. This test was conducted using variance inflation factors [59], and the VIF function of the R-package car [60] was applied to the full linear model excluding the random effect (i.e. macaque ID). VIF values greater than 10 indicate that the two predictors are highly collinear [59]. We removed from the final model the predictor, in each set of highly correlated predictors, that had the lowest likelihood ratio after running the model [59]. Furthermore, Cook’s distance was measured in order to identify if there were outliers, which could influence the model [59]. To be accepted, Cook’s distance must be lower than 1 [59]; this was always the case in the present study. In addition, we checked that assumptions of the normal distribution of the data and the homogeneity of the residuals were not violated, by visually inspecting a q-q plot where the residuals were plotted against fitted values [59]. The model was accepted if the q-q plot was close to linear. However when the assumption of normality was not met, i.e. the q-q plot was not linear, the dependent variable was Log_10_ transformed in order to improve the normal distribution of the residuals. P-values for each test were derived using the functions pvals.fnc and aovlmer.fnc of the R package language [61] and were based on Markov Chain Monte Carlo sampling [53].

## Results

### Comparison of the different health measures between groups

#### Births and deaths

In 2011, one female in each group was not observed with a live new-born infant, and thus the percentage of females with new-born infants was similarly high in the two groups - 90% in Tourist Group (TG), and 85.7% in Green Group (GG). The percentage of females observed with a new-born infant was lower in TG than in GG in 2012 (TG = 45.5%; GG = 100%). In 2013, one female from TG did not give birth, while all females in GG did (TG = 91.7%; GG = 100%).

During the present study period (March to December 2012), in TG, one miscarriage was seen, one adult male died and two other adults (one male and one female) disappeared and were presumed dead, thus 12.5% of adults died or were presumed dead in TG; in GG no such events occurred.

#### Lameness and disease symptoms

Over the whole study period, 29% of study animals in TG (4 males and 1 female) and 9% in GG (one male) presented long-lasting lameness, and this lasted throughout the whole study period for all of these animals.

Individuals from both groups were occasionally seen coughing and sneezing during the study period. There were three periods when most of the individuals in the TG were seen coughing and sneezing heavily, numerous times per day: 6^th^–24^th^ March, 23^rd^June–7^th^ July, and 22^nd^–29^th^ December 2012. In GG, four individuals were reported coughing and sneezing heavily on one day (8^th^ August 2012). Over the study period, 32 occurrences of diarrhoea were reported for TG (0.0085 diarrheal symptoms per animal per observation day) and only one for GG (0.0011 diarrheal symptoms per animal per observation day); therefore the rate of occurrence was nearly eight times higher in TG.

#### Scars and injuries

The majority of individuals from both groups presented some visible scars. There was no significant difference in the number of visible scars between groups for females (Mann-Whitney test: N = 15, U = -0.255, P = 0.799) or for males (Independent t test: N = 13, t = 2.131, P = 0.059), although there was a trend towards TG males having more visible scars than GG males.

There was no significant difference between groups in the number of injuries over the study period for females (Mann-Whitney test: N = 15, U = 12.000, P = 0.075) or males (Mann-Whitney test: N = 13, U = 13.000, P = 0.301).

#### Body size

**Fig 3:**
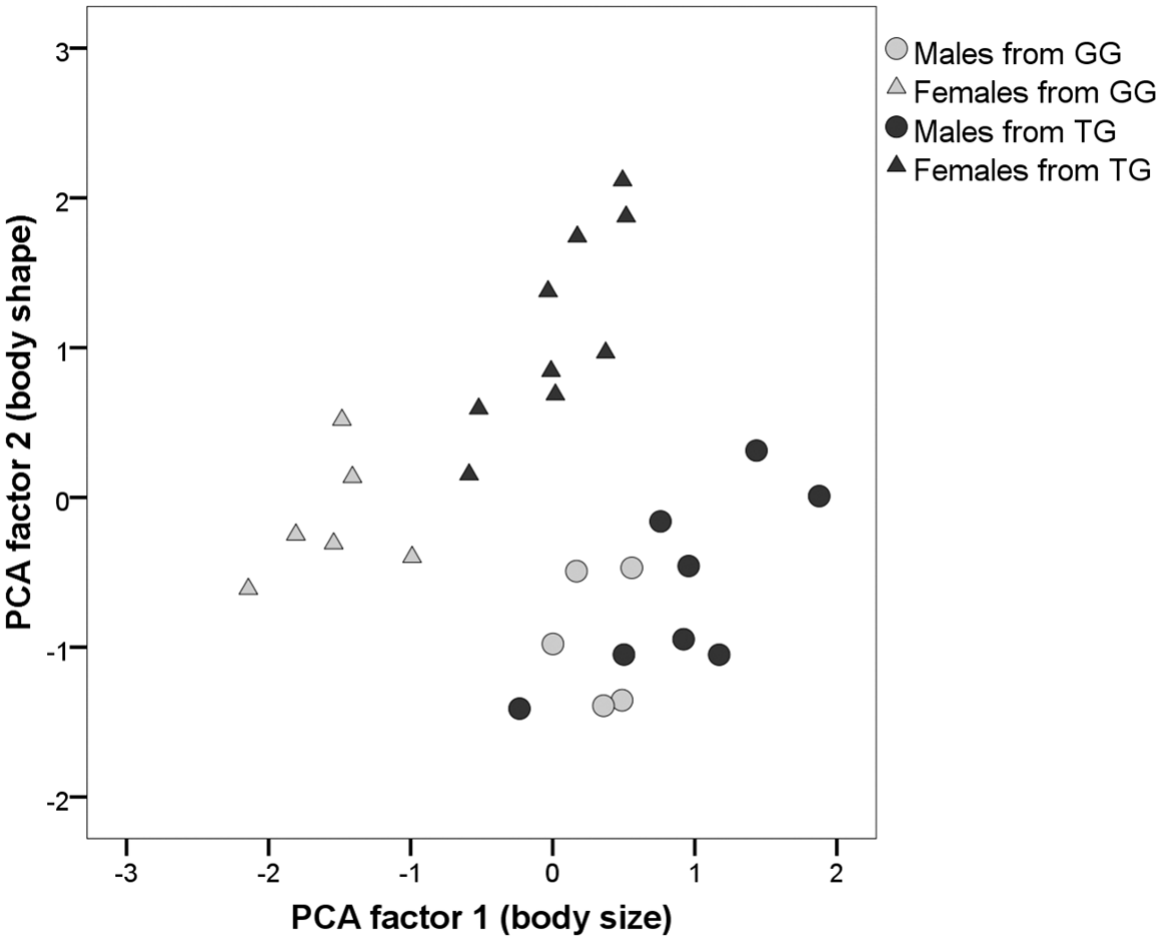
Scatterplot showing scores for PCA 1 (body size) and PCA 2 (body shape) for each individual from TG and GG. Each point represents an individual, categorised by group and sex.

**Fig 4:**
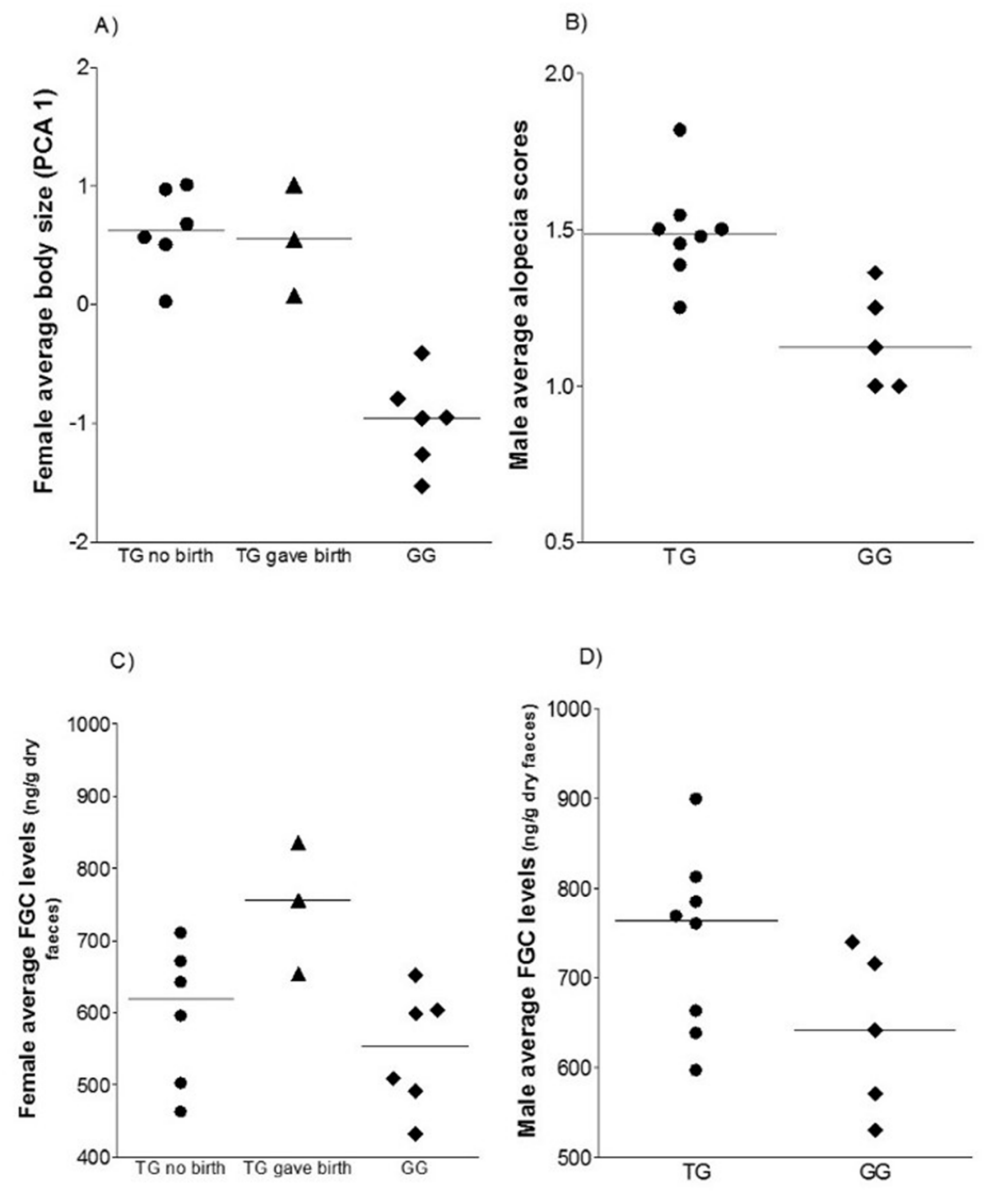
Scatterplots showing the comparison between TG and GG for: A) Female average body size, B) Male average alopecia scores, C) Female average FGC levels, D) Male average FGC levels. Symbols represent each individual and lines median values.

Over the whole study period, and in all months, TG females had significantly larger body size (PCA 1 scores, Fig 3) than GG females (Independent t tests whole period: TG who gave birth vs. GG females: N = 9, t = 7.469, P<0.001; TG females who did not give birth vs. GG females: N = 12, t = 4.949, P = 0.002; for monthly results see Table A and Fig A in S1 File) (Fig 4A). By contrast, there was no significant difference between groups for males across the whole study period (Independent t test: N = 13, t = 2.082, P = 0.062), although there was a trend towards males in the TG being larger in size, and TG males were significantly larger July-September (see Table B and Fig B in S1 File).

#### Coat quality

There was no significant difference between groups in coat quality across the whole study period, nor in any month, either for females (Independent t test: provisioned females gave birth vs. non-provisioned females: N = 9, t = -1.660, P = 0.141; provisioned females did not give birth vs. non-provisioned females: N = 12, t = -0.811, P = 0.436) or for males (Independent t test: N = 13, t = 0.144, P = 0.888; for monthly results see Tables C and D, Figs C and D in S1 File).

#### Alopecia

There was no significant difference between groups in female alopecia scores across the whole study period, nor in any month (Independent t test: provisioned females gave birth vs. non-provisioned females: N = 9, t = 0.036, P = 0.973; provisioned females did not give birth vs. non-provisioned females: N = 12, t = -0.155, P = 0.880; for monthly results see Table E and Fig E in S1 File). However, there was a significant difference between groups across the whole study period (Independent t test: N = 13, t = 2.304, P = 0.042), with TG males having more alopecia (Fig 4B), though no difference in any individual month (see Table F and Fig F in S1 File).

#### Faecal glucocorticoid (FGC) metabolite levels

TG females who gave birth had higher FGC levels than GG females (all of whom gave birth) across the whole study period (Independent t test: N = 9, t = 3.943, P = 0.006, Fig 4C) and in March, June and July (see Table G and Fig G in S1 File). There was no significant difference between TG females that did not give birth and GG females across the whole study period, (Independent t test: N = 12, t = 0.966, P = 0.357), though TG females had higher levels in June and July (see Table G and Fig G in S1 File). TG males had significantly higher FGC levels than GG males across the whole study period (Independent t test: N = 13, t = 2.701, P = 0.021, Fig 4D) and in April and June (see Table H and Fig H in S1 File).

### Relationships between health measures and intensity of provisioning in TG

For complete GLMM results, see Tables I and J in S1 File.

#### Body size

Females and males were bigger when the intensity of provisioning (the percentage of total feeding scans spent feeding on human food) was higher (females: N = 99, estimate = 0.01, ±SE = 0.003, t = 3.183, P = 0.004; males: N = 86, estimate = 0.01, ±SE = 0.004, t = 2.588, P = 0.013).

#### Coat quality

There was a significant positive association between female coat quality scores and the intensity of provisioning (N = 99, estimate = -0.007, ±SE = 0.003, t = -2.504, P = 0.016); in males, there was no relationship between these variables (N = 86, estimate = 0.00, ±SE = 0.003, t = -0.159, P = 0.887).

#### Alopecia

There was no relationship between alopecia scores and intensity of provisioning for either females (N = 99, estimate = -0.059, ±SE = 0.137, t = -0.43, P = 0.632) or males (N = 86, estimate = 0.002, ±SE = 0.003, t = 0.753, P = 0.435).

#### Faecal glucocorticoid (FGC) metabolite levels

Females’ FGC levels were not linked with intensity of provisioning (N = 262, estimate = 0.001, ±SE = 0.001, t = 1.82, P = 0.081); however males’ FGC levels were higher when levels of provisioning were higher (N = 309, estimate = 0.001, ±SE = 0.001, t = 2.33, P = 0.021).

## Discussion

We explored the impacts of tourist provisioning on the health of Barbary macaques in Ifrane National Park, Morocco. Overall, results indicate that feeding by tourists has negative impacts on the health of these animals, and that some such impacts may be sex-specific. Formal statistical comparison between the groups revealed a larger body size for provisioned females (but not males), higher physiological stress (FGC levels) for provisioned males and females, and more alopecia for provisioned males (but not females). No differences were found between the two groups in relation to number of scars and injuries, or in coat quality. Within the provisioned group, higher levels of provisioning were associated with increased body size in both sexes, better coat quality in females and higher physiological stress levels in males.

### Descriptive data: Births, deaths, lameness and disease symptoms

The comparisons between groups for births, mortality rates, lameness and disease symptoms are only descriptive in nature, and could not be explored statistically due to sample size issues. Nevertheless, they are at least indicative of some effects of provisioning, and we feel that reporting of such data is important to facilitate future comparative and/or meta-analyses exploring tourism impacts.

Although in 2011 and 2013, there was not a major difference between groups in the number of births, in 2012 the birth rate was markedly lower in the provisioned than the non-provisioned group, with 45% and 100% of females giving birth respectively. One confirmed death, two disappearances of adult monkeys and one miscarriage occurred in the provisioned group, but no such events occurred in the non-provisioned group over the same period. The one animal found dead was examined post-mortem by a local vet, who suggested it may have died from food poisoning, possibly linked with provisioning. Only a few studies have suggested that the death of an animal may directly result from provisioning in wild settings [62, 63], and post-mortem examinations of wild animals are rarely conducted.

29% of study animals in the provisioned group, but only 9% in the non-provisioned group, showed signs of lameness, which might be linked to intraspecific aggression during provisioning, or to injuries related to harmful garbage (e.g. opened metal cans, broken glass, or sharp plastic bottles) that is very common on the ground at the tourist site. Disease symptoms seemed more frequent in the provisioned group, even considering that more full days were spent with this group. Disease transmission between people and animals is a serious concern when humans interact with wildlife, particularly during provisioning when physical contact between people and animals is often observed [64, 65]. For example, we often observed at the site tourists cracking peanut shells in their mouth and giving them to the monkeys, or drinking water from a bottle and then handing it to the monkeys to drink from, greatly increasing the risks of disease transmission by fluid exchange.

### Scars and injuries

No difference between groups was seen in terms of the number of visible scars or injuries. Although previous studies indicate that higher rates of aggression towards conspecifics occur during provisioning in a range of species [15, 66–68], including Barbary macaques [69], this may reflect non-contact rather than physically injurious aggression (e.g. rhesus macaques [70]). Moreover, Barbary macaques have an ‘egalitarian’ dominance style [71], and display more non-physical aggression rather than physical forms [72], which may explain why there was no significant difference between provisioned and non-provisioned groups in the numbers of scars or injuries per individual.

### Body size

Provisioned females, though not males, had larger body size than non-provisioned animals of the same sex throughout the year, and for both males and females in the provisioned group body size was positively related to the intensity of provisioning. A number of studies have suggested that the poor diet of tourist-provisioned animals leads to them becoming overweight, and thus exposed to the associated health risks [9, 18], although we currently lack an understanding of what represents optimally healthy body size and weight for wild animals. Nevertheless, our findings suggest that provisioning is pushing female Barbary macaques above what is a normal body size, and therefore presumably a normal body weight. Similarly, [73] found that provisioning through garbage raiding led to a markedly greater increase in body mass among female than male yellow baboons. These studies highlight the importance of exploring possible effects of food supplementation separately by sex, and potentially by age class.

### Body condition—coat quality and alopecia

High levels of provisioning were related to better coat quality for provisioned females but not for provisioned males, suggesting the females may be more energetically or nutritionally stressed. Poor diet has been associated with poor coat quality in a number of domesticated or livestock species, such as dogs [74] and sheep [75]; for wild animals, however, provisioning may improve body condition by increasing energy intake [76]. Interestingly, no overall difference in coat quality was seen between provisioned and non-provisioned troops for either sex. Barbary macaques have a flexible diet, and may be able to cope with seasonal variation in food availability and nutritional requirements by diversifying their diet [35], which may explain this finding.

Males in the provisioned troop had significantly more alopecia than males in the non-provisioned troop; no such difference was found for females. These results are in line with findings in Japanese macaques, where individuals from provisioned groups had more alopecia than those from non-provisioned groups [77]. Hair loss may be a serious issue because it compromises the efficient protection provided by an animal’s fur [78]; this may be particularly problematic for species like Barbary macaques that experience extreme climate variation and can suffer significant mortality in winter [79]. Stress is one of the main factors leading to alopecia [47, 80], and provisioning might cause psychological and physiological stress due to the increased risk of aggression with conspecifics and/or tourists [27, 69].

### Physiological stress levels

Tourist provisioning appears to be stressful for Barbary macaques: across the whole study period and in some months in spring and summer, animals in the provisioned troop had higher physiological stress levels than those in the non-provisioned troop; additionally, for males in the provisioned group, stress levels were related to the intensity of provisioning. The link between provisioning and stress levels may be explained by an increase in the intensity of intraspecific competition due to the clumped nature of food resources received from tourists [69], or by the occurrence of agonistic interactions with tourists [27]. A study at the same site found evidence that in the provisioned group, males were found in closer proximity to tourists than were females, and males also had more interactions with tourists [81]. These effects may be alleviated by the spreading of food resources and by prohibiting the giving of food by hand (such provisioning was often observed at the site); these changes have been implemented in a number of primate tourism settings, for example with Tibetan macaques [82], and Japanese macaques [83].

Since physiological stress levels have been found to be negatively related to body condition [84, 85], and survival [84, 86], the evidence presented here suggests that tourist provisioning may be having a negative impact on the welfare and fitness of Barbary macaques. Nevertheless, the present results must be interpreted with caution as threshold levels over which physiological stress becomes deleterious for wild Barbary macaques are not known; a long term study would be needed to assess whether elevated FGC concentrations lead to negative fitness outcomes.

### Interpreting the results of health measures

Determining the full impacts of tourist provisioning on wild animal health is challenging, due to a lack of understanding of optimum indices or ranges for the health measures employed, and of knowledge about threshold levels, under or over which individual fitness might be detrimentally affected. This is particularly true when using non-invasive health measures. Impacts of provisioning may, additionally, only manifest themselves in the long term [8] or be related to other factors such as age. Results of this study must be also interpreted with caution, due to the relatively small sample size in terms of number of animals, the lack of replication at the levels of groups, and our inability to exclude completely potential differences in habitat quality between the study sites. Moreover, it is important to acknowledge the potential effect of the difference in group size between the study troops. For instance, the larger group size in the provisioned group might be associated with higher levels of intra-group aggression, with increased physiological stress levels as a result. However, group size might itself increase in relation to provisioning, and this variable may therefore help to explain (rather than confound) findings. Nevertheless, when considering the overall results for the different health measures in the present study, it seems reasonable to suggest that some regulation of tourist provisioning of Barbary macaques at the study site would be beneficial.

### A framework for the costs and benefits of provisioning

**Fig 5:**
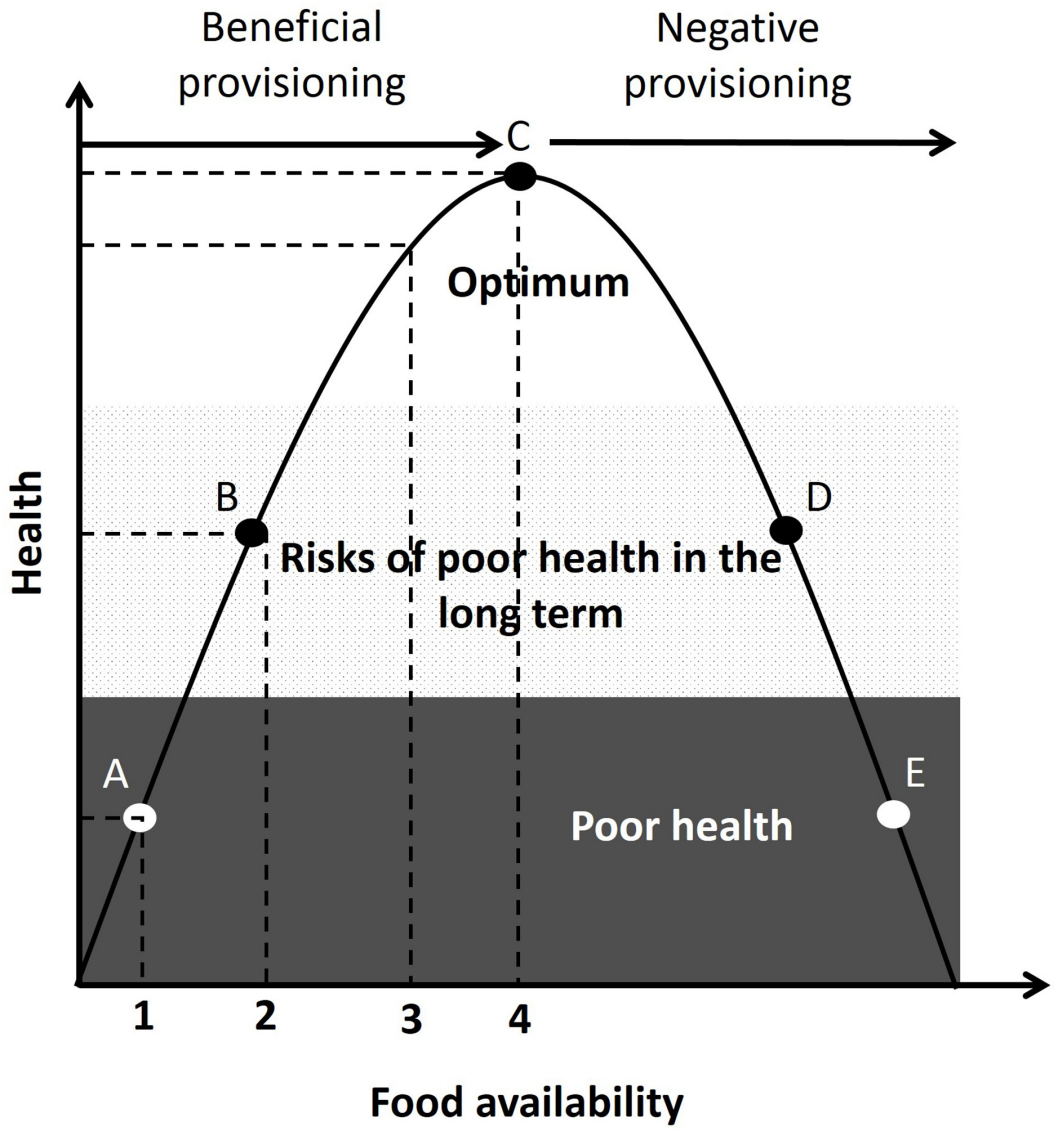
Framework for the effects of food availability—the sum of both natural and provisioned food—on the health of wild animals. The numbers (1–4) represent the different levels of natural food availability, and the letters the associated health outcomes—**A**: Animals in poor health which threatens immediate survival, and for which provisioning might improve survival. **B**: Animals in relatively poor health, which decreases chance of survival in the long term, but can be improved by provisioning. **C**: Animals with optimal health. **D**: Animals in relatively poor health condition, due to excessive food intake with lower chance of survival in long term, but can be improved by reducing provisioning. **E**: Animals in poor health which threatens immediate survival, but chance of survival might be improved by drastically reducing provisioning.

In Fig 5, we provide a framework for helping to visualise the potential costs and benefits of provisioning. A key assumption of this framework is that the relationship between health and total food availability—the summation of both natural food and provisioning—is an inverted U shape. This type of relationship, called hormesis [87], has been shown to describe the link between body mass index and mortality rates in humans [88]. The framework considers different levels of natural food availability (with variation being due, for example, to seasonal or annual effects) and the potential consequences of provisioning on the health of wild animals. In scenario 1, natural food availability is so low that animals are under imminent threat for their survival, and provisioning in this case may be highly recommended. In scenario 2, natural food availability is low, increasing the risks of long term health problems or premature death, and provisioning may be beneficial. In scenario 3, natural food availability is in the range required for good health, and provisioning may have only limited beneficial impacts or no impact. Finally, when natural food availability is at a level that leads to optimal health (scenario 4), provisioning may have negative impacts on health.

Clearly, more data on health and particularly on the fitness impacts (positive and negative) of provisioning in wild populations are now needed to parameterise this framework. Nevertheless, it provides a tool that may spur future research (for example to identify and quantify the different costs and benefits of provisioning), or facilitate management decisions on whether provisioning may be acceptable or not, according to its impacts on the health of wild animals. It highlights the importance of assessing natural food availability as well as optimal health requirements for the animal (e.g. energy intake/energy expenditure) in order to estimate whether provisioning may be an acceptable practice. This framework may also help those involved in conservation management considering the regulation of tourist provisioning, and could also be adapted to other health measures such as stress and disease risks.

## Conclusion

Overall, our study contributes to an understanding of the impacts of tourist provisioning on the health of wild animals. The results highlight the importance of using a range of health measures to provide a comprehensive picture of the impacts of tourist provisioning on animal health. Our findings also highlight, however, the limitations in assessing health of wild animals, as optimum ranges and thresholds for health measures are generally unknown. Provisioning is a part of wildlife tourism at many tourist sites [1], and if wildlife tourism is to be beneficial for the conservation of endangered species, understanding the full impacts of such behaviour is crucial. In Morocco, wildlife tourism is a growing industry and tourist provisioning is often the main activity at tourist sites where wild animals, including Barbary macaques, can be seen. Our study provides evidence that tourist provisioning has overall negative impacts on the health of the macaques, and suggests that regulating such feeding provisioning may be key to making wildlife tourism more sustainable and ‘eco-friendly’.

## Supporting Information

S1 Data Monthly and daily data recorded on the provisioned group (TG) and the non-provisioned group (GG). (XLSX)

S1 File Additional result details. Monthly comparisons between groups (Tables A-H, Figs A-H), and GLMM results (Tables I and J).
(PDF)

## References

[1] Orams MB. Feeding wildlife as a tourism attraction: a review of issues and impacts. Tourism management. 2002; 23: 281–93.

[2] Kurita H. Provisioning and tourism in free-ranging Japanese macaques In: Russon A.E., and Wallis J., eds. Primate tourism: a tool for conservation?, Cambridge University Press; 2014 pp. 44–56.

[3] Dubois S, Fraser D. A Framework to Evaluate Wildlife Feeding in Research, Wildlife Management, Tourism and Recreation. Animals. 2013; 3: 978–94. 10.3390/ani3040978 26479747 PMC4494361

[4] Newsome D, Rodger K. Feeding of wildlife: an acceptable practice in ecotourism? In: Ballantyne R., and Packer J., eds. International handbook on ecotourism, Edward Elgar Publishing limited, UK; 2013 pp. 520.

[5] Higginbottom K, Tribe A. Contributions of wildlife tourism to conservation. Wildlife tourism: Impacts, management and planning; 2004 pp. 99–123.

[6] Ballantyne R, Packer J, Hughes K. Tourists' support for conservation messages and sustainable management practices in wildlife tourism experiences. Tourism Management. 2009; 30: 658–64.

[7] Maljković A, Côté IM. Effects of tourism-related provisioning on the trophic signatures and movement patterns of an apex predator, the Caribbean reef shark. Biological Conservation. 2011; 144: 859–65.

[8] Knapp CR, Hines KN, Zachariah TT, Perez-Heydrich C, Iverson JB, Buckner SD, et al Physiological effects of tourism and associated food provisioning in an endangered iguana. Conservation Physiology. 2013; 1: cot032. 27293616 10.1093/conphys/cot032 PMC4806617

[9] Perez CE, Bensusan KJ. Upper Rock nature reserve: a management and action plan. Gibraltar Ornithological and Natural History Society; 2005 pp. 297.

[10] Semeniuk CA, Bourgeon S, Smith SL, Rothley KD. Hematological differences between stingrays at tourist and non-visited sites suggest physiological costs of wildlife tourism. Biological Conservation. 2009 142: 1818–29.

[11] Foroughirad V, Mann J. Long-term impacts of fish provisioning on the behavior and survival of wild bottlenose dolphins. Biological Conservation. 2013; 160: 242–9.

[12] Wikelski M, Cooke SJ. Conservation physiology. Trends in Ecology & Evolution. 2006; 21: 38–46. 16701468 10.1016/j.tree.2005.10.018

[13] Deem SL, Karesh WB, Weisman W. Putting theory into practice: wildlife health in conservation. Conservation Biology. 2001; 15: 1224–33.

[14] Zhao QK, Deng ZY. Dramatic consequences of food handouts to *Macaca thibetana* at Mount Emei, China. Folia Primatologica. 1992; 58: 24–31.

[15] Semeniuk CA, Rothley KD. Costs of group-living for a normally solitary forager: effects of provisioning tourism on southern stingrays *Dasyatis americana*. Marine Ecology Progress Series. 2008; 357: 271.

[16] Borg C, Majolo B, Qarro M, Semple S. A comparison of body size, coat condition and endoparasite diversity of wild Barbary macaques exposed to different levels of tourism. Anthrozoös. 2014; 27: 49–63.

[17] Hamada Y, Watanabe T, Iwamoto M. Physique index for Japanese macaques (*Macaca fuscata*): age change and regional variation. Anthropological Science. 1996; 104: 305–23.

[18] Fa JE. The Barbary macaques_ A case study in conservation. Plenum Press, New-York; 1984.

[19] Mokdad AH, Ford ES, Bowman BA, Dietz WH, Vinicor F, Bales VS, et al Prevalence of obesity, diabetes, and obesity-related health risk factors, 2001. Jama. 2003; 289: 76–9. 12503980 10.1001/jama.289.1.76

[20] Sapolsky RM. Some pathogenic consequences of tourism for non-human primates In: Russon A.E. and Wallis J., eds. Primate tourism: a tool for conservation?, Cambridge University Press; 2014 pp. 147–155.

[21] Jungheim E. Obesity and Fertility. Springer 2015.

[22] Jolly A. Coat condition of ringtailed lemurs, *Lemur catta* at Berenty Reserve, Madagascar: I. Differences by age, sex, density and tourism, 1996–2006. American Journal of Primatology. 2009; 71: 191–8. 10.1002/ajp.20647 19051320

[23] Amo L, López P, Martín J. Nature-based tourism as a form of predation risk affects body condition and health state of *Podarcis muralis* lizards. Biological Conservation. 2006; 13: 402–9.

[24] Goldberg TL, Gillespie TR, Rwego IB, Wheeler E, Estoff EL, Chapman CA. Patterns of gastrointestinal bacterial exchange between chimpanzees and humans involved in research and tourism in western Uganda. Biological Conservation. 2007; 135: 511–7.

[25] Rwego IB, Isabirye-Basuta GI, Gillespie TR, Goldberg TL. Gastrointestinal bacterial transmission among humans, mountain gorillas, and livestock in Bwindi Impenetrable National Park, Uganda. Conservation Biology. 2008; 22: 1600–7. 10.1111/j.1523-1739.2008.01018.x 18717695

[26] Fowler GS. Behavioral and hormonal responses of Magellanic penguins (*Spheniscus magellanicus*) to tourism and nest site visitation. Biological Conservation. 1999; 90: 143–9.

[27] Maréchal L, Semple S, Majolo B, Qarro M, Heistermann M, MacLarnon A. Impacts of tourism on anxiety and physiological stress levels in wild male Barbary macaques. Biological Conservation. 2011; 144: 2188–93.

[28] Shutt K, Heistermann M, Kasim A, Todd A, Kalousova B, Profosouva I, et al Effects of habituation, research and ecotourism on faecal glucocorticoid metabolites in wild western lowland gorillas: Implications for conservation management. Biological Conservation. 2014; 172: 72–9.

[29] Ménard N, Vallet D. Behavioral responses of Barbary macaques (*Macaca sylvanus*) to variations in environmental conditions in Algeria. American Journal of Primatology. 1997; 43: 285–304. 9403094 10.1002/(SICI)1098-2345(1997)43:4<285::AID-AJP1>3.0.CO;2-T

[30] Mouna M, Ciani AC. Distribution and demography of Barbary macaque (*Macaca sylvanus*) in the wild In: Hodges J.K. and Cortes J., eds. The Barbary macaque: Biology, management and conservation, Nottingham: Nottingham University Press; 2006 pp. 239–256.

[31] van Lavieren E, Wich SA. Decline of the Endangered Barbary macaque *Macaca sylvanus* in the cedar forest of the Middle Atlas Mountains, Morocco. Oryx. 2010; 44: 133–8.

[32] Butynski TM, Cortes J, Waters S, Fa JE, Hobbelink ME, van Lavieren E, et al. *Macaca sylvanus* In: IUCN 2010. IUCN Red List of Threatened Species. Version 2010.4. 2008. Available from www.iucnredlist.org> (19.02.13).

[33] Haut-Commissariat des Eaux et Forêts et à la Lutte contre la Désertification. Conservation Action Plan for the Barbary macaque (*Macaca sylvanus*) in Morocco. 2012.

[34] Majolo B, McFarland R, Young C, Qarro M. The effect of climatic factors on the activity budgets of Barbary macaques (*Macaca sylvanus*). International Journal of Primatology. 2013; 34: 500–14.

[35] Ménard N. Ecological plasticity of Barbary macaques (*Macaca sylvanus*). Evolutionary Anthropology: Issues, News, and Reviews. 2002; 11: 95–100.

[36] Altmann J. Observational study of behavior: sampling methods. Behaviour. 1974; 49: 227–66. 4597405 10.1163/156853974x00534

[37] Breuer T, Robbins MM, Boesch C. Using photogrammetry and color scoring to assess sexual dimorphism in wild western gorillas (*Gorilla gorilla*). American Journal of Physical Anthropology. 2007; 134: 369–82. 17657788 10.1002/ajpa.20678

[38] Kurita H, Suzumura T, Kanchi F, Hamada Y. A photogrammetric method to evaluate nutritional status without capture in habituated free-ranging Japanese macaques (*Macaca fuscata*): a pilot study. Primates. 2012; 53: 7–11. 10.1007/s10329-011-0280-4 22057793

[39] Rohlf FJ. Morphometrics at SUNY Stony Brook (online). 2010. Retrieved from: 'http://life.bio.sunysb.edu/morph/soft-dataacq.html' (18/02/2013).

[40] Jolicoeur P, Mosimann JE. Size and shape variation in the painted turtle. A principal component analysis. Growth. 1960; 24: 339–54. 13790416

[41] Honess P, Gimpel J, Wolfensohn S, Mason G. Alopecia scoring: the quantitative assessment of hair loss in captive macaques. Alternatives to laboratory animals: ATLA. 2005; 33: 193–206. 16180975 10.1177/026119290503300308

[42] Hodges JK, Heistermann M. Field endocrinology: monitoring hormonal changes in free-ranging primates In: Setchell J.M., and Curtis D.J., eds. Field and laboratory methods in primatology: A practical guide, Cambridge: Cambridge University Press; 2003 pp. 282–294.

[43] Möhle U, Heistermann M, Dittami J, Reinberg V, Hodges JK. Patterns of anogenital swelling size and their endocrine correlates during ovulatory cycles and early pregnancy in free-ranging Barbary macaques (*Macaca sylvanus*) of Gibraltar. American Journal of Primatology. 2005; 66: 351–68. 16104035 10.1002/ajp.20161

[44] Ganswindt A, Palme R, Heistermann M, Borragan S, Hodges JK. Non-invasive assessment of adrenocortical function in the male African elephant (*Loxodonta africana*) and its relation to musth. General and Comparative Endocrinology. 2003; 134: 156–66. 14511986 10.1016/s0016-6480(03)00251-x

[45] Heistermann M, Palme R, Ganswindt A. Comparison of different enzymeimmunoassays for assessment of adrenocortical activity in primates based on fecal analysis. American Journal of Primatology. 2006; 68: 257 16477600 10.1002/ajp.20222

[46] Heistermann M. Non-invasive monitoring of endocrine status in laboratory primates: methods, guidelines and applications. Advances in Science and Research. 2010; 5: 1–9.

[47] Novak MA, Meyer JS. Alopecia: possible causes and treatments, particularly in captive nonhuman primates. Comparative Medicine. 2009; 59: 18 19295051 PMC2703143

[48] Beehner JC, Nguyen N, Wango EO, Alberts SC, Altmann J. The endocrinology of pregnancy and fetal loss in wild baboons. Hormones and Behavior. 2006; 49: 688–99. 16487522 10.1016/j.yhbeh.2005.12.016

[49] Hoffman CL, Higham JP, Heistermann M, Coe CL, Prendergast BJ, Maestripieri D. Immune function and HPA axis activity in free-ranging rhesus macaques. Physiology & Behavior. 2011; 104: 507–14. 21635909 10.1016/j.physbeh.2011.05.021 PMC3133459

[50] Cristóbal-Azkarate J, Maréchal L, Semple S, Majolo B, MacLarnon A. Metabolic strategies in free ranging male Barbary macaques: evidence from faecal measurement of thyroid hormone. Biology Letters. 2016; 12: 20160168 10.1098/rsbl.2016.0168 27095269 PMC4881361

[51] Young C, Majolo B, Heistermann M, Schülke O, Ostner J. Male mating behaviour in relation to female sexual swellings, socio-sexual behaviour and hormonal changes in wild Barbary macaques. Hormones and Behavior. 2013; 63: 32–9. 10.1016/j.yhbeh.2012.11.004 23146839

[52] Rice WR. Analyzing tables of statistical tests. Evolution. 1989: 223–5. 28568501 10.1111/j.1558-5646.1989.tb04220.x

[53] Baayen RH. Analyzing linguistic data: A practical introduction to statistics using R. Cambridge University Press; 2008.

[54] R" Development" Core" Team. R: a language and environment for statistical computing. 2011. Available from http://www.R.project.org.

[55] Bates D, Maechler M. lme4: Linear mixed-effects models using S4 classes. In: R package version 0.999375–37. 2010.

[56] Peduzzi P, Concato J, Kemper E, Holford TR, Feinstein AR. A simulation study of the number of events per variable in logistic regression analysis. Journal of clinical epidemiology. 1996; 49: 1373–9. 8970487 10.1016/s0895-4356(96)00236-3

[57] Bolker BM, Brooks ME, Clark CJ, Geange SW, Poulsen JR, Stevens MH et al Generalized linear mixed models: a practical guide for ecology and evolution. Trends in Ecology & Evolution. 2009; 24: 127–35. 19185386 10.1016/j.tree.2008.10.008

[58] Mundry R, Nunn CL. Stepwise model fitting and statistical inference: turning noise into signal pollution. The American Naturalist. 2009; 173: 119–23. 10.1086/593303 19049440

[59] Field A, Miles J, Field Z. Discovering statistics using R. London: Sage Publications; 2012.

[60] Fox J, Weisberg HS. An R companion to applied regression, 2nd ed Sage Publications Inc., Thousand oaks; 2010.

[61] Baayen RH. languageR: Data sets and functions with “Analyzing Linguistic Data: A practical introduction to statistics”. R package version 0.953. 2010. Available from http://CRAN.R-project.org/package=language.

[62] Cunningham-Smith P, Colbert DE, Wells RS, Speakman T. Evaluation of human interactions with a provisioned wild bottlenose dolphin (*Tursiops truncatus*) near Sarasota Bay, Florida, and efforts to curtail the interactions. Aquatic Mammals. 2006; 32: 346–56.

[63] de Sá Alves LC, Andriolo A, Orams MB, de Freitas Azevedo A. Resource defence and dominance hierarchy in the boto (*Inia geoffrensis*) during a provisioning program. Acta Ethologica. 2013; 16: 9–19.

[64] Fuentes A. Human culture and monkey behavior: assessing the contexts of potential pathogen transmission between macaques and humans. American Journal of Primatology. 2006; 68: 880–96. 16900502 10.1002/ajp.20295

[65] Muehlenbein MP, Wallis J. Considering risks of pathogen transmission associated with primate-based tourism In Russon A.E. and Wallis J., eds. Primate tourism: a tool for conservation?, Cambridge University Press; 2014 pp. 278–292.

[66] Hill DA. Effects of provisioning on the social behaviour of Japanese and rhesus macaques: implications for socioecology. Primates. 1999; 40: 187–98. 10.1007/BF02557710 23179540

[67] Ram S, Venkatachalam S, Sinha A. Changing social strategies of wild female bonnet macaques during natural foraging and on provisioning. Current Science-Bangalore. 2003; 84: 780–90.

[68] Hsu MJ, Kao CC, Agoramoorthy G. Interactions between visitors and Formosan macaques (*Macaca cyclopis*) at Shou-Shan Nature Park, Taiwan. American Journal of Primatology. 2009; 71: 214–22. 10.1002/ajp.20638 19051313

[69] Majolo B, van Lavieren E, Maréchal L, MacLarnon A, Qarro M, Marvin G, et al Out of Asia: The singular case of the Barbary macaque In: Radhakrishna S, Sinha A, Huffman M, eds. The macaque connection: Cooperation and conflict between humans and macaques. Springer-Verlag; 2013 pp. 167–183.

[70] Southwick CH. An experimental study of intragroup agonistic behavior in rhesus monkeys (*Macaca mulatta*). Behaviour. 1967; 28: 182–209. 4959568 10.1163/156853967x00235

[71] Thierry B, Aureli F. Barbary but not barbarian: social relations in a tolerant macaque In: Hodges J.K. and Cortes J.. eds. The Barbary macaque: biology, management and conservation. Nottingham: Nottingham University Press; 2006 pp. 29–45.

[72] De Waal F, Luttrell LM. Toward a comparative socioecology of the genus *Macaca*: different dominance styles in rhesus and stumptail monkeys. American Journal of Primatology. 1989; 19: 83–109. 10.1002/ajp.1350190203 31964014

[73] Altmann J, Schoeller D, Altmann SA, Muruthi P, Sapolsky RM. Body size and fatness of free-living baboons reflect food availability and activity levels. American Journal of Primatology. 1993; 30: 149–61. 10.1002/ajp.1350300207 31937018

[74] Marsh KA. Measuring skin and coat condition in healthy dogs. Waltham focus; 1999; 9: 31.

[75] Khan MJ, Abbas A, Ayaz M, Naeem M, Akhter MS, Soomro MH. Factors affecting wool quality and quantity in sheep. African Journal of Biotechnology. 2012; 11: 13761–6.

[76] Robb GN, McDonald RA, Chamberlain DE, Bearhop S. Food for thought: supplementary feeding as a driver of ecological change in avian populations. Frontiers in Ecology and the Environment. 2008; 6: 476–84.

[77] Zhang P. A non-invasive study of alopecia in Japanese macaques *Macaca fuscata*. Current Zoology. 2011; 57: 26–35.

[78] Tregear RT. Hair density, wind speed, and heat loss in mammals. Journal of Applied Physiology. 1965 7 1;20:796–801. 5838737 10.1152/jappl.1965.20.4.796

[79] McFarland R, Majolo B. Coping with the cold: predictors of survival in wild Barbary macaques, *Macaca sylvanus*. Biology Letters. 2013; 9: 20130428 10.1098/rsbl.2013.0428 23804292 PMC3730655

[80] Steinmetz HW, Kaumanns W, Dix I, Heistermann M, Fox M, Kaup FJ. Coat condition, housing condition and measurement of faecal cortisol metabolites–a non-invasive study about alopecia in captive rhesus macaques (*Macaca mulatta*). Journal of Medical Primatology. 2006; 35: 3–11. 16430489 10.1111/j.1600-0684.2005.00141.x

[81] Maréchal L. Investigating primate tourism in Morocco using a multidisciplinary approach. PhD thesis, University of Roehampton; 2015.

[82] Usui R, Sheeran LK, Li JH, Sun L, Wang X, Pritchard AJ, et al Park Rangers’ Behaviors and Their Effects on Tourists and Tibetan Macaques (*Macaca thibetana*) at Mt. Huangshan, China. Animals. 2014; 4: 546–61. 10.3390/ani4030546 26480324 PMC4494317

[83] Knight J. Herding monkeys to paradise: how macaque troops are managed for tourism in Japan. Leiden: Bill; Biggleswade: Extenza Turpin; 2011.

[84] Romero LM, Wikelski M. Corticosterone levels predict survival probabilities of Galapagos marine iguanas during El Nino events. Proceedings of the National Academy of Sciences. 2001; 98: 7366–70. 10.1073/pnas.131091498 PMC34674 11416210

[85] Cabezas S, Blas J, Marchant TA, Moreno S. Physiological stress levels predict survival probabilities in wild rabbits. Hormones and Behavior. 2007; 51: 313–20. 17258747 10.1016/j.yhbeh.2006.11.004

[86] Pride RE. High faecal glucocorticoid levels predict mortality in ring-tailed lemurs (*Lemur catta*). Biology Letters. 2005; 1: 60–3. 17148128 10.1098/rsbl.2004.0245 PMC1965197

[87] Calabrese EJ, Baldwin LA. Defining hormesis. Human & Experimental Toxicology. 2002; 21: 91–7. 12102503 10.1191/0960327102ht217oa

[88] Allison DB, Faith MS, Heo M, Kotler DP. Hypothesis concerning the U-shaped relation between body mass index and mortality. American Journal of Epidemiology. 1997; 146: 339–49. 9270413 10.1093/oxfordjournals.aje.a009275

