# Supplementary material for: Assessing the Effects of Tourist Provisioning on the Health of Wild Barbary Macaques in Morocco
Source: PLoS One. 2016 May 20;11(5):e0155920. doi: 10.1371/journal.pone.0155920 (PMC4874683; doi:10.1371/journal.pone.0155920)
Supplement: S1 File — Monthly comparisons between groups (Tables A-H, Figs A-H), and GLMM results (Tables I and J). (PDF) [file pone.0155920.s002.pdf]

Supplementary information:

S1 File. Additional result details

Comparison of body size between groups per month

Females

Table A. Monthly comparison of PCA 1 scores (body size) between groups for females. Of the reported test statistics, t is associated with the independent t-test and U with Mann-Whitney test. Values in bold were significant after sequential Bonferroni corrections.

	March	April	May	June	July	August	September	October	November	December	
t/U	t=3.811	t=7.152	t=3.643	t=2.792	t=3.714	U=0.000	t=4.082	t=4.702	t=4.046	t=5.865	TG gave birth vs. GG
P value	**0.007**	**<0.001**	**0.008**	**0.027**	**0.008**	**0.02**	**0.006**	**0.002**	**0.050**	**0.001**	
t/U	t=3.356	t=5.316	t=3.434	t=3.235	t=8.116	U=0.000	t=6.718	t=7.157	t=6.626	t=10.134	TG no birth vs. GG
P value	**0.007**	**<0.001**	**0.006**	**0.009**	**<0.001**	**0.004**	**<0.001**	**<0.001**	**<0.001**	**<0.001**	

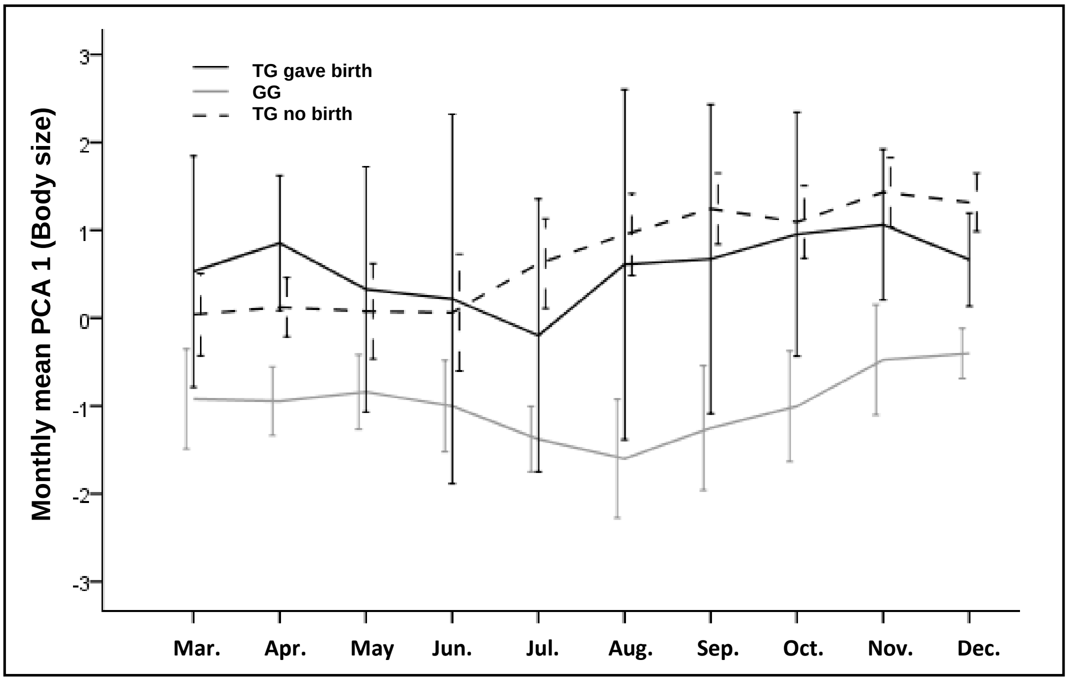

Fig A. Plots of seasonal variation of mean PCA 1 scores (body size) for TG and GG females. Error bars indicate 95% confidence intervals. Please refer to Table A1 for results of statistical analyses.

Males

Table B. Monthly comparison of PCA 1 scores (body size) between groups for males. Values in bold are significant after sequential Bonferroni correction.

	March	April	May	June	July	August	September	October	November	December
t	0.107	-0.502	0.238	2.122	4.815	5.354	4.200	2.576	1.737	1.013
P value	0.917	0.626	0.816	0.057	**0.001**	**<0.001**	**0.001**	0.026	0.113	0.335

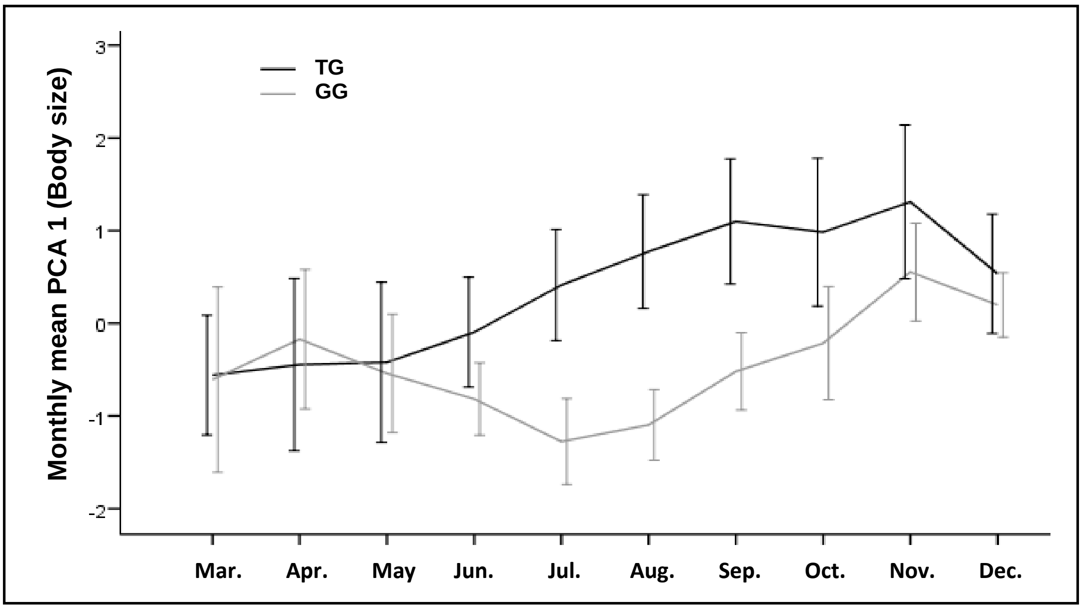

Fig B. Plots of seasonal variation of mean PCA 1 scores (body size) for TG and GG males. Error bars indicate 95% confidence intervals. Please refer to Table A2 for results of statistical analyses.

Comparison of coat quality between groups per month

Females

Table C. Monthly comparison of TG and GG female coat quality scores. Of the reported test statistics, t is associated with the independent t-test and U with Mann-Whitney test.

	March	April	May	June	July	August	September	October	November	December	
t/U	t=-1.594	U=8.000	t=0.333	t=0.509	U=0.000	U=6.500	U=3.000	U=7.000	t=-0.972	U=9.000	TG gave birth vs. GG
P value	0.155	0.783	0.749	0.626	0.017	0.009	0.248	0.583	0.363	1.000	
t/U	t=1.830	U=3.000	t=-1.296	t=-1.754	U=4.500	U=13.000	U=13.000	U=15.000	t=-0.200	U=16.500	TG no birth vs. GG
P value	0.097	0.012	0.224	0.110	0.026	0.006	0.159	0.604	0.845	0.784	

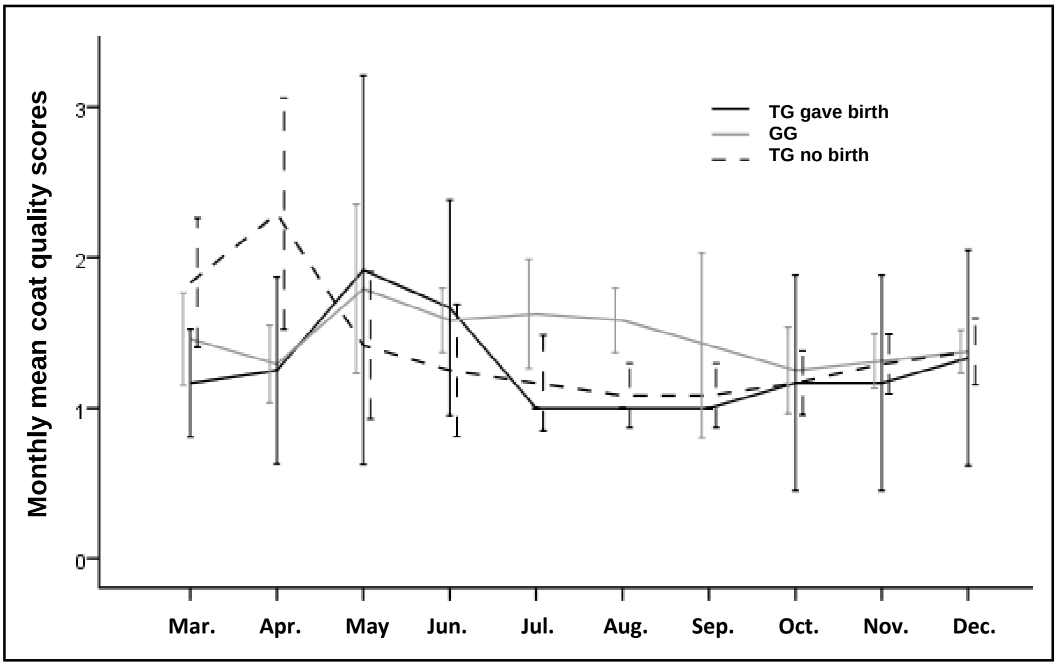

Fig C. Plots of seasonal variation of mean coat quality scores for TG and GG females. Error bars indicate 95% confidence intervals. Please refer to Table A3 for results of statistical analyses.

Males

Table D. Monthly comparison of male coat quality between TG and GG groups. Of the reported test statistics, t is associated with the independent t-test and U with Mann-Whitney test.

	March	April	May	June	July	August	September	October	November	December
t/U	U=12.500	t=-0.363	U=20.000	U=8.000	t=-1.320	U=19.000	U=17.000	t=0.108	t=0.743	t=0.611
P value	0.263	0.724	1.000	0.062	0.214	0.876	0.627	0.916	0.474	0.555

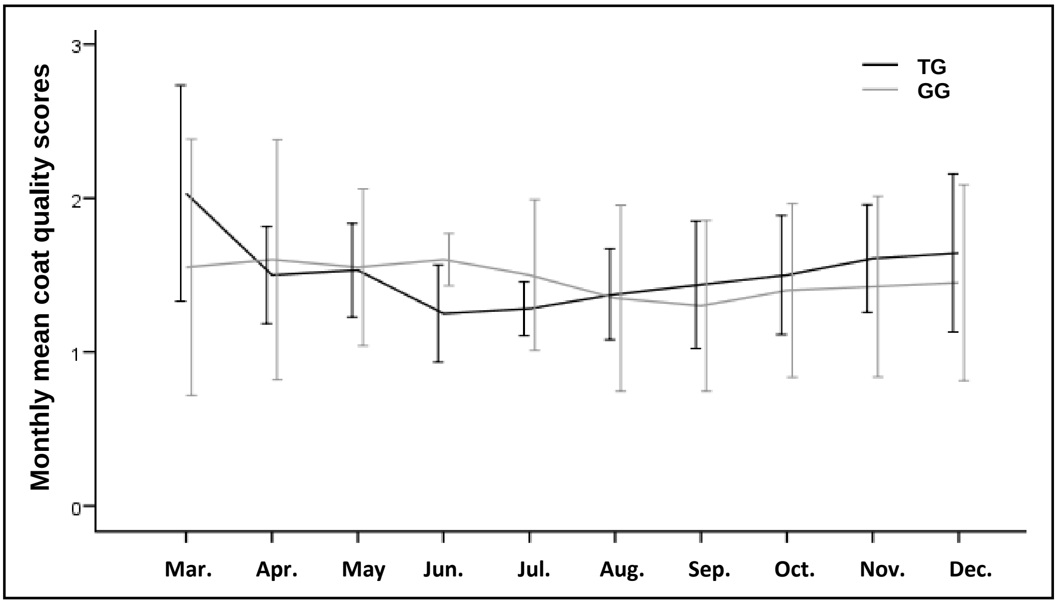

Fig D. Plots of seasonal variation of mean coat quality for TG and GG males. Error bars indicate 95% confidence intervals.

Comparison of alopecia between groups per month

Females

Table E. Monthly comparison of female alopecia scores between groups. Of the reported test statistics, t is associated with the independent t-test and U with Mann-Whitney test.

	March	April	May	June	July	August	September	October	November	December	
t/U	t=0.189	U=8.000	t=3.311	U=6.000	U=5.000	U=6.500	U=3.500	t=-1.080	t=-0256	t=-0.306	TG gave birth vs. GG
P value	0.855	0.785	0.013	0.394	0.283	0.496	0.378	0.316	0.805	0.769	
t/U	t=1.771	U=15.500	t=-0.591	U=12.000	U=6.500	U=13.000	U=15.000	t=0.500	t=0.070	t=0.000	TG no birth vs. GG
P value	0.107	0.666	0.568	0.140	0.047	0.388	0.361	0.628	0.946	1.000	

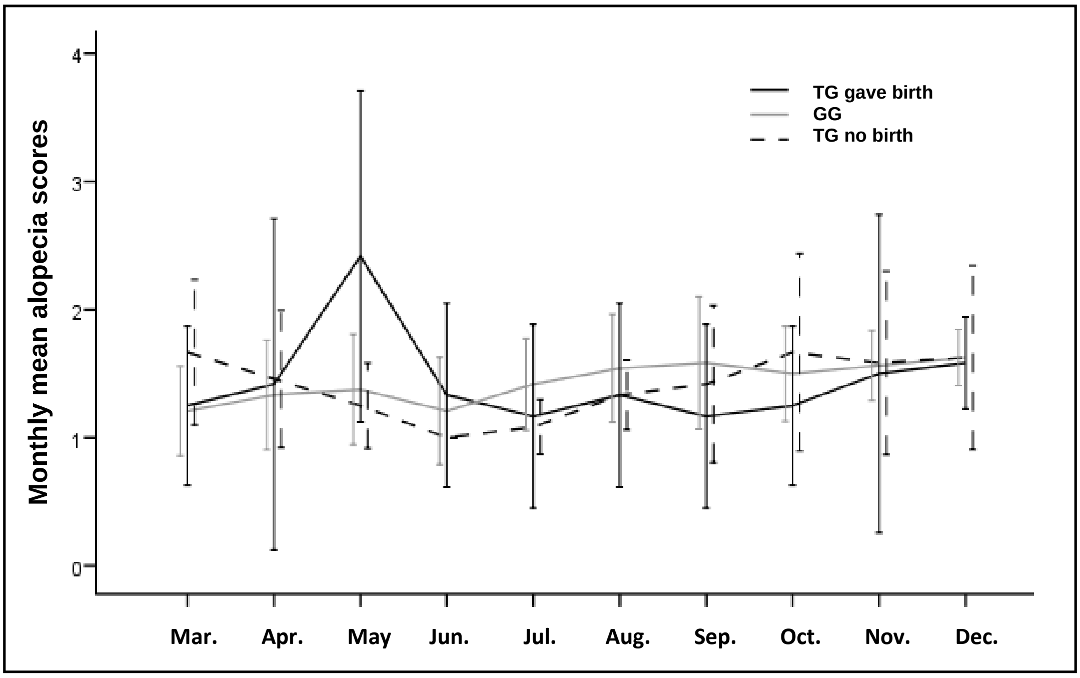

Fig E. Plots of seasonal variation of mean alopecia scores for TG and GG females. Error bars indicate 95% confidence intervals.

Males

Table F. Monthly comparison of male alopecia scores between groups. Of the reported test statistics, t is associated with the independent t-test and U with Mann-Whitney test.

	March	April	May	June	July	August	September	October	November	December
t/U	U=2.500	U=7.500	U=10.000	U=11.000	U=19.000	U=10.000	U=18.000	U=13.500	t=0.438	t=2.083
P value	0.007	0.036	0.109	0.139	0.816	0.073	0.747	0.300	0.670	0.064

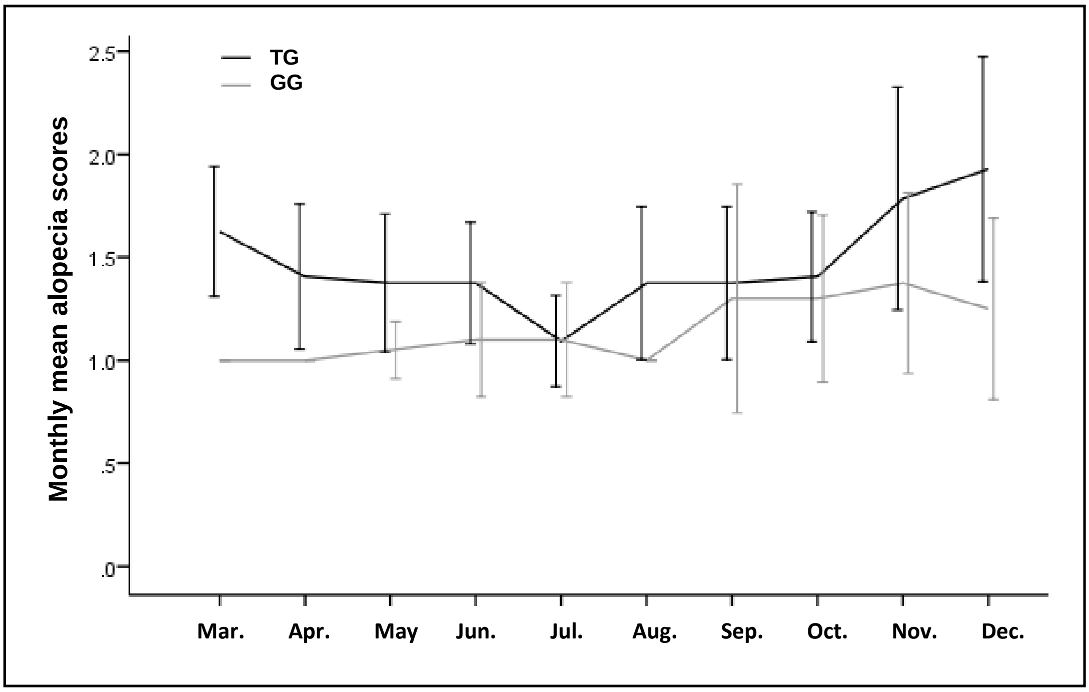

Fig F. Plots of seasonal variation of mean alopecia scores for TG and GG males. Error bars indicate 95% confidence intervals.

Comparison of FGC levels between groups per month

Females

Table G. Monthly comparison of female FGC levels (ng/g dry faeces) between groups. Of the reported test statistics, t is associated with the independent t-test and U with Mann-Whitney test. Values in bold are significant after sequential Bonferroni corrections.

	March	April	May	June	July	August	September	October	November	December	
t/U	t=4.116	t=2.8	t=2.973	t=4.806	t=5.966	t=0.811	t=0.321	t=1.931	U=5.000	t=-0.716	TG gave birth vs. GG
P value	**0.004**	0.027	0.021	**0.002**	**0.001**	0.444	0.759	0.095	0.302	0.497	
t/U	t=-1.624	t=0.891	t=1.346	t=4.455	t=3.716	t=0.108	t=0.312	t=1.198	U=13.000	t=-0.714	TG no birth vs. GG
P value	0.135	0.394	0.208	**0.001**	**0.004**	0.916	0.761	0.258	0.423	0.492	

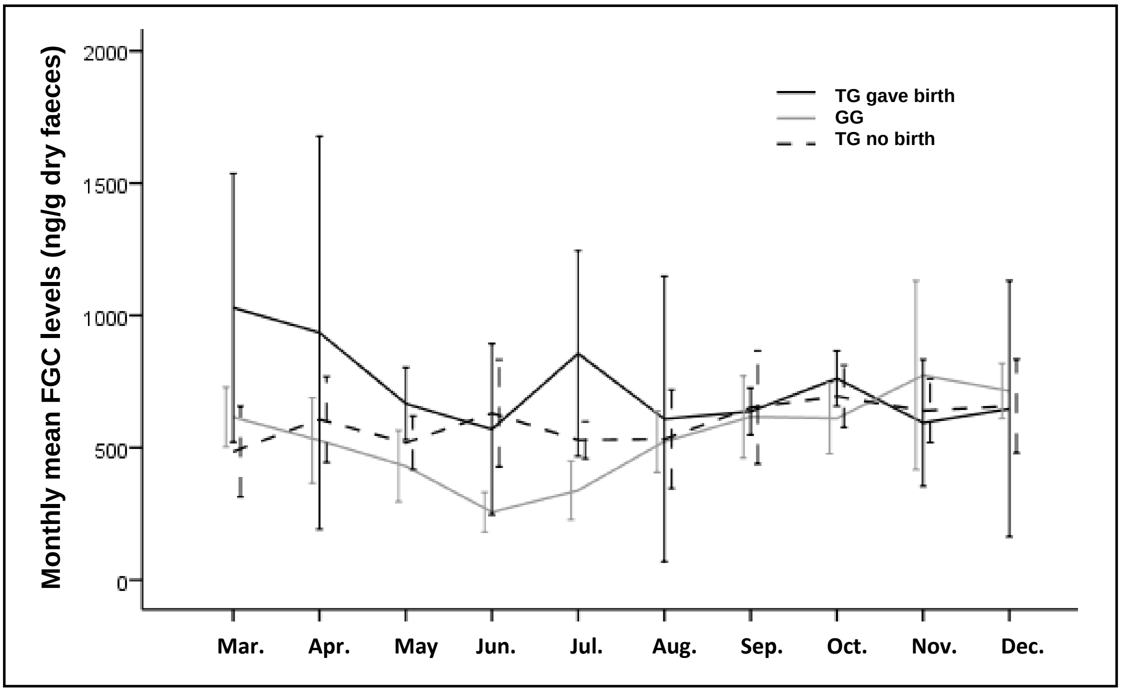

Fig G. Plots of seasonal variation of mean FGC levels for TG and GG females. Error bars indicate 95% confidence intervals. Please refer to Table A9 for results of statistical analyses.

Males

Table H. Monthly comparison of male FGC levels (ng/g dry faeces) between groups. Of the reported test statistics, t is associated with the independent t-test and U with Mann-Whitney test. Values in bold are significant after sequential Bonferroni corrections.

	March	April	May	June	July	August	September	October	November	December
t/U	t=1.699	t=4.264	U=4.000	t=4.651	t=2.885	t=1.410	t=0.857	t=0.883	t=-0.655	t=-2.288
P value	0.117	**0.001**	0.019	**0.001**	0.015	0.186	0.410	0.396	0.527	0.045

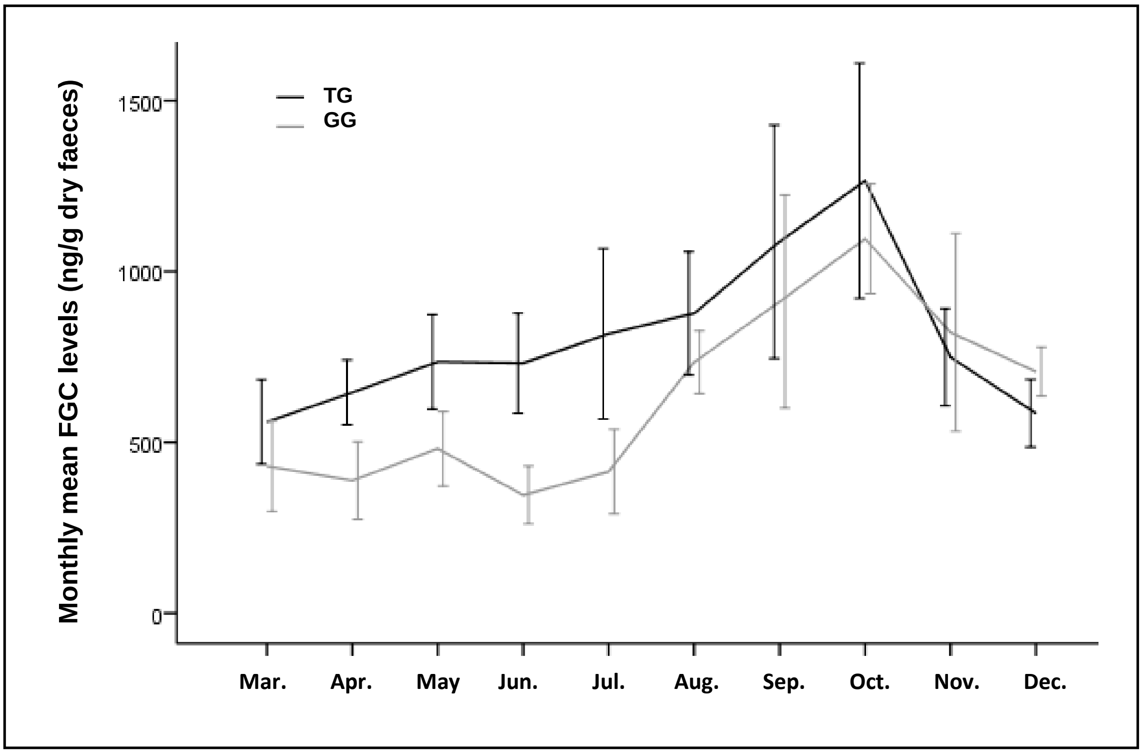

Fig H. Plots of seasonal variation of mean TG and GG male FGC levels. Error bars indicate 95% confidence intervals. Please refer to Table A10 for results of statistical analyses.

Relationships between intensity of provisioning and the different health measures within the provisioned group: Full GLMM tables

Females

Table I. Results of GLMM testing the relationships between the different health measures and amount of provisioning for the females of the Tourist Group over the 10 month study period.

Model Response	Null vs. Full model			Predictor variables									
				**Provisioning**	Birth vs. No Birth	Rainfall	Rank	Social season					
								Birth vs Mating	Birth vs. Post Birth	Birth vs. Pre Birth	Mating vs. Post Birth	Mating vs. Pre Birth	Post Birth vs. Pre Birth
Body Size	N	99	Estimate	0.01	0.22	0.08	0.01	0.83	0.65	0.75	-0.18	-0.08	0.10
	df	7	± SE	0.00	0.20	0.01	0.12	0.16	0.19	0.26	0.13	0.19	0.18
	χ2	59.89	t value	3.18	1.09	5.95	0.06	5.03	3.34	2.93	-1.41	-0.43	0.57
	P	***<0.001***	P_{MCMC}	***0.004***	*0.34*	***<0.001***	*0.93*	***<0.001***	***0.001***	***0.01***	*0.20*	*0.70*	*0.61*
Coat quality	N	99	Estimate	-0.01	-0.18	-0.01	0.01	-0.81	-0.75	-0.60	0.06	0.21	0.14
	df	7	± SE	0.00	0.08	0.01	0.04	0.14	0.17	0.22	0.12	0.17	0.16
	χ2	36.38	t value	-2.50	-2.10	-1.16	0.33	-5.65	-4.40	-2.73	0.53	1.23	0.92
	P	***<0.001***	P_{MCMC}	***0.02***	*0.09*	*0.26*	*0.80*	***<0.001***	***<0.001***	***0.01***	*0.61*	*0.24*	*0.39*
Alopecia	N	99	Estimate	-0.06	0.00	0.01	-0.03	0.06	-0.03	0.19	-0.08	0.13	0.22
	df	7	± SE	0.14	0.00	0.01	0.07	0.17	0.20	0.27	0.14	0.20	0.19
	χ2	5.76	t value	-0.43	-0.11	0.97	-0.43	0.32	-0.13	0.71	-0.59	0.67	1.14
	P	*0.568*	P_{MCMC}	*0.63*	*0.90*	*0.36*	*0.72*	*0.77*	*0.90*	*0.51*	*0.59*	*0.52*	*0.29*
FGC levels	N	262	Estimate	0.00	0.20	-0.01	0.10	0.15	-0.12	-0.03	-0.28	-0.19	0.09
	df	7	± SE	0.00	0.07	0.01	0.03	0.09	0.12	0.12	0.10	0.11	0.08
	χ2	27.13	t value	1.82	2.86	-0.71	2.96	1.75	-1.08	-0.27	-2.79	-1.72	1.20
	P	***<0.001***	P_{MCMC}	*0.08*	***0.03***	*0.49*	***0.03***	*0.09*	*0.29*	*0.80*	***0.01***	*0.09*	*0.23*

Males

Table J. Results of GLMM testing the relationships between the different health measures and amount of provisioning for the males of the Tourist Group over the 10 month study period.

Model Response	Null vs. Full model			Predictor variables								
				**Provisioning**	Rainfall	Rank	Social season					
							Birth vs Mating	Birth vs. PostBirth	Birth vs. PreBirth	Mating vs. PostBirth	Mating vs. PreBirth	PostBirth vs. PreBirth
Body Size	N	86	Estimate	0.01	0.10	-0.35	1.35	1.50	0.92	0.14	-0.43	-0.57
	df	6	± SE	0.00	0.02	0.16	0.26	0.33	0.39	0.21	0.29	0.25
	χ2	52.12	t value	2.59	4.40	-2.29	5.17	4.52	2.37	0.69	-1.50	-2.28
	P	***<0.001***	P_{MCMC}	***0.01***	***<0.001***	***0.02***	***<0.001***	***<0.001***	***0.03***	*0.53*	*0.17*	***0.04***
Coat quality	N	86	Estimate	0.00	-0.02	0.41	0.33	-0.18	0.29	-0.50	-0.04	0.46
	df	6	± SE	0.00	0.01	0.05	0.16	0.16	0.18	0.12	0.12	0.13
	χ2	47.53	t value	-0.16	-1.82	7.92	2.00	-1.10	1.61	-4.22	-0.34	3.57
	P	***<0.001***	P_{MCMC}	*0.89*	*0.09*	***<0.001***	*0.06*	*0.30*	*0.12*	***<0.001***	*0.74*	***0.002***
Alopecia	N	86	Estimate	0.00	0.00	0.06	0.46	0.10	0.24	-0.36	-0.22	0.14
	df	6	± SE	0.00	0.01	0.05	0.13	0.18	0.14	0.18	0.14	0.20
	χ2	14.93	t value	0.75	-0.37	1.29	3.48	0.58	1.65	-1.97	-1.63	0.68
	P	***0.02***	P_{MCMC}	*0.44*	*0.69*	*0.39*	***0.00***	*0.57*	*0.10*	*0.06*	*0.12*	*0.52*
FGC levels	N	309	Estimate	0.00	0.03	-0.02	0.16	0.44	0.17	0.27	0.00	-0.27
	df	6	± SE	0.00	0.01	0.04	0.09	0.11	0.12	0.09	0.10	0.08
	χ2	32.71	t value	2.33	3.84	-0.57	1.80	4.11	1.43	3.19	0.01	-3.53
	P	***<0.001***	P_{MCMC}	***0.02***	***<0.001***	*0.61*	*0.07*	***<0.001***	*0.17*	***0.002***	*1.00*	***<0.001***
